# Incorporation of Hydrophilic Macrocycles Into Drug-Linker Reagents Produces Antibody-Drug Conjugates With Enhanced *in vivo* Performance

**DOI:** 10.3389/fphar.2022.764540

**Published:** 2022-06-17

**Authors:** Nick Evans, Ruslan Grygorash, Paul Williams, Andrew Kyle, Terrence Kantner, Ravindra Pathak, XiaoBo Sheng, Fabio Simoes, Hiteshri Makwana, Ricardo Resende, Elena de Juan, Alan Jenkins, David Morris, Aurelie Michelet, Frances Jewitt, Felicity Rudge, Nicolas Camper, Anaïs Manin, William McDowell, Martin Pabst, Antony Godwin, Mark Frigerio, Matthew Bird

**Affiliations:** Abzena Ltd., Babraham Research Campus, Babraham, United Kingdom

**Keywords:** antibody drug conjugate (ADC), crown ether, cyclodextrin, *in vivo*, xenograft

## Abstract

Antibody-drug conjugates (ADCs) have begun to fulfil their promise as targeted cancer therapeutics with ten clinical approvals to date. As the field matures, much attention has focused upon the key factors required to produce safe and efficacious ADCs. Recently the role that linker-payload reagent design has on the properties of ADCs has been highlighted as an important consideration for developers. We have investigated the effect of incorporating hydrophilic macrocycles into reagent structures on the *in vitro* and *in vivo* behavior of ADCs. *Bis*-sulfone based disulfide rebridging reagents bearing Val-Cit-PABC-MMAE linker-payloads were synthesized with a panel of cyclodextrins and crown ethers integrated into their structures *via* a glutamic acid branching point. Brentuximab was selected as a model antibody and ten ADCs with a drug-to-antibody ratio (DAR) of 4 were prepared for biological evaluation. *In vitro*, the ADCs prepared showed broadly similar potency (range: 16–34 pM) and were comparable to Adcetris^®^ (16 pM). *In vivo*, the cyclodextrin containing ADCs showed greater efficacy than Adcetris^®^ and the most efficacious variant (incorporating a 3′-amino-α-cyclodextrin component) matched a 24-unit poly(ethylene glycol) (PEG) containing comparator. The ADCs bearing crown ethers also displayed enhanced *in vivo* efficacy compared to Adcetris^®^, the most active variant (containing a 1-aza-42-crown-14 macrocycle) was superior to an analogous ADC with a larger 24-unit PEG chain. In summary, we have demonstrated that hydrophilic macrocycles can be effectively incorporated into ADC reagent design and offer the potential for enhanced alternatives to established drug-linker architectures.

## Introduction

The concept of antibody-drug conjugates (ADCs) as targeted cancer therapeutics combines the unique targeting properties of a monoclonal antibody to deliver a high potency of a cytotoxin attached via an appropriate linker. As the number of ADCs entering the clinic and achieving regulatory approval continues to grow, there remains a concerted research effort to explore alternative drug-linker designs with the aim of enhancing ADC efficacy and safety. A wide array of structural options for the linker component connecting the antibody to the cytotoxic drug exist. Recent attention has focused on the use of site-specific conjugating linkers to improve ADC homogeneity and a variety of both cleavable and non-cleavable linkers have been employed to maximize toxin delivery to tumor cells (Frigerio and Kyle, 2017; Zhou, 2017; Dal Corso et al., 2019).

Recent research has focused on the inclusion of polymeric portions within ADC linker structure to overcome some of the inherent issues surrounding ADC design such as hydrophobicity, aggregation, instability, insufficient drug-loading and reduced circulatory half-life. Typically, the polymers have been amphiphilic in nature, being water-soluble, synthetic and substantially non-antigenic with particular focus on polyalkylene oxides such as poly(ethylene glycol) (PEG). Since the first PEGylated protein was approved in 1990, PEG has become one of the most widely used polymers in biopharmaceutical applications with numerous products entering clinical trials and receiving marketing approval (Veronese and Mero, 2008; Turecek et al., 2016). PEG features within two of the currently approved ADCs, sacituzumab govitecan (Trodelvy^®^) and loncastuximab tesirine (Zynlonta^®^), and its utility within ADC reagents has been widely investigated, mainly to improve reagent solubility and to reduce aggregation of the final ADC. The correlation between ADC hydrophobicity and accelerated plasma clearance, particularly for conjugates with high drug-loading, has highlighted the value of incorporating a hydrophilic polymer moiety to mitigate the hydrophobic effects of drug-linkers (Hamblett et al., 2004; Lyon et al., 2015). Recent studies have shown that reagent design, and particularly the positioning of the PEG within the ADC architecture, is an essential consideration when developing the optimal ADC. Some groups describe the use of linear PEG spacers which separates the drug moiety from the antibody (Tiberghien et al., 2016; Bryden et al., 2018; Yang et al., 2019). Others have highlighted an improved reagent format whereby the PEG exists as a pendant molecule which is attached to the reagent *via* a branching linker (Zhao et al., 2008; Lyon et al., 2015; Burke et al., 2017; Shao et al., 2018; Shao et al., 2020). Our approach has been to use glutamic acid as a branching point to suitably position a PEG chain and a cleavable linker-payload and these reagent structures have been shown to produce efficacious DAR 4, 6 and 8 ADCs (Pabst et al., 2017). ADCs with branched formats have shown improvements in pharmacokinetic (PK) behavior and enhanced *in vivo* efficacy when compared to ADCs where PEG exists as a spacer segment between the antibody and drug molecules (Lyon et al., 2015; Burke et al., 2017; Pabst et al., 2017). A common feature with these pendant structures is the polymer tethered to the linker has a single point of attachment leaving a free end group in solution. The unattached end of the polymer chain in PEG is typically a methoxy group. It has been suggested that when PEG is arranged in a pendant configuration, it allows for more effective masking of the hydrophobic drug-linker portion of the ADC (Lyon et al., 2015).

Alternative polymers to PEG have also been integrated into ADC reagent design. Submonomer solid phase synthesis has allowed the production of monodisperse polysarcosine (PSAR) oligomers containing 6–24 repeat units (Viricel et al., 2019). Trastuzumab-glucuronide-MMAE based ADCs containing PSAR in branched (*n* = 6, 12, 18 and 24 units) and spacer (*n* = 12 units) formats were compared *in vivo* alongside structurally analogous ADCs with either no polymer or PEG (*n* = 12 units) in the branched format. ADCs containing the branched reagent structure had improved PK and efficacy compared to the ADCs with no polymer and where the spacer configuration was employed. The branched PSAR(12u) ADC was also found to be more efficacious than a PEG(12u) containing variant. Polyacetal polymers, as part of the Fleximer^®^ technology platform, have also found utility in the development of ADCs with significantly higher drug-to-antibody ratios (DAR ≥20) compared to more common conjugation approaches, which typically produce DARs between 2 to 4 (Yurkovestskiy et al., 2015). Other polymeric reagent architectures have been reported (Zhang et al., 2017; Marcinkowska et al., 2019; Shi et al., 2020), however PEG, with its long-established history and application in the enhancement of biopharmaceuticals, remains at the forefront in the design of more efficacious ADCs. In contrast to the active research interest in the use of polymers in ADC reagents, there are no reports of ADC reagents incorporating pendant macrocyclic moieties where both ends of the polymer chain are tethered to the reagent linker and the pharmacological impact of introducing such a structural constraint.

Macrocyclic molecules, comprising twelve or more atoms in a ring structure, are long established in medicine as drug molecules and drug delivery vehicles. Cyclodextrins, macrocycles constructed of cyclic arrangements of glucopyranose units, have been extensively investigated in drug delivery applications (Challa et al., 2005; Loftsson et al., 2005; Jansook et al., 2018; Kim et al., 2020). Three naturally occurring cyclodextrins (α-, β- and γ-), containing 6, 7 or 8 glucopyranose repeats connected in cycles *via* α-1,4-glycosidic linkages respectively, are known. As drug carriers, cyclodextrins have been applied to a wide range of biologics including genes, peptides, proteins and oligonucleotides (Irie and Uekama, 1999; Redenti et al., 2001; Ortiz Mellet et al., 2011; Haley et al., 2020). Small molecules, including cytotoxic agents, however, have been the predominant research focus, with over 35 cyclodextrin containing pharmaceutical products gaining market approval (Loftsson and Brewster, 2010; Kurkov and Loftsson, 2013; Gidwani and Vyas, 2015). Crown ethers, cycles consisting of oxyethylene repeat units, are macrocyclic analogues of PEG which have been shown to have a wide variety of activities, ranging from ion complexation and micelle formation (Gokel et al., 1988) to antimicrobial activity (Febles et al., 2016). Substantial research demonstrating the drug delivery potential of crown ethers has also been reported (Jansen et al., 2002; Muzzalupo et al., 2007; Chehardoli and Bahmani, 2019).

With cyclodextrins and crown ethers established in (bio)pharmaceutical applications, the aim of this study was to determine whether these macrocycles may be used as hydrophilic substitutes for the polymeric element present within ADC reagent structures to generate an efficacious drug molecule. In the present study, we demonstrate the effect that integrating such hydrophilic macrocycles into drug-linker reagent structures has on the *in vitro* and *in vivo* properties of a series of homogeneous DAR 4 ADCs. The ADCs investigated incorporate the anti-CD30 antibody brentuximab, with the prodrug Val-Cit-PABC-MMAE and either cyclodextrin or crown ether macrocycles linked *via* a glutamic acid branching point. Conjugation to the antibody was achieved using an established *bis*-sulfone based disulfide rebridging linker, previously shown to produce stable and homogeneous DAR 4 ADCs (Badescu et al., 2014; Bryant et al., 2015). Analogous ADCs with linear PEGs were prepared as comparators and the biological performance of the conjugates produced were benchmarked against the clinically approved ADC, brentuximab vedotin (Adcetris^®^).

## Materials and Methods

### ADC Reagent Synthesis

Reagents **1a-f**, **2a-b** and **3a-b** were prepared using published synthetic methods (Godwin, 2017; Godwin et al., 2017). Preparative silica gel and C18 reverse phase chromatography purifications were conducted on a Reveleris^®^ X2 Flash Chromatography System.

### ADC Production

Brentuximab-MMAE ADCs were prepared as previously described (Godwin, 2017; Godwin et al., 2017). Briefly, brentuximab (in 20 mM sodium phosphate, pH 7.5, 150 mM NaCl, 20 mM EDTA) was reduced at 5 mg/ml antibody concentration with TCEP (1.5 equivalents per disulfide) for 1 h at 40°C. Reduced antibody solutions were cooled to 22°C and diluted to 4.21 mg/ml with 20 mM sodium phosphate, pH 7.5, 150 mM NaCl, 20 mM EDTA. Conjugation reagents (**1a-f**, **2a-b** and **3a-b**) in MeCN were added (5% v/v MeCN, 1.4 equivalents per disulfide) to the reduced antibody. Conjugation reactions were incubated for16-20 h and subsequently quenched by the addition of 50 mM *N*-acetyl-l-cysteine (3 mM final concentration). The crude reactions were then purified by hydrophobic interaction chromatography (HIC) using ToyoPearl Phenyl-650S columns, as described previously (Bird et al., 2020). Fractions containing DAR 4 ADC were pooled and buffer exchanged into PBS. Antibody concentration was determined by Bradford assay (Expedeon). DAR 4 was determined by HIC-HPLC, a more quantitative characterisation than SDS-PAGE or UV-vis (see Bird et al., 2020)

### *In vitro* Cytotoxicity Assay

*In vitro* potency was evaluated in Karpas-229 cells using a CellTiter-Glo^®^ luminescence assay (Promega). Karpas-299 cells were cultured in RPMI-1640 medium (Life Technologies®) supplemented with 10% fetal bovine serum, 100 U/mL penicillin and 100 μg/ml streptomycin. Karpas-299 cells were counted using disposable Neubauer counting chambers and cell density was adjusted to 0.25 × 10^4^ cells per well (50 μL/well). Cells were incubated for 24 h at 37°C with 5% CO_2_. The cells were treated by addition of ADCs **4a-f**, **5a-b** and **6a-b**, Adcetris^®^ (Takeda) or MMAE (Concortis) dilution series (50 µL/well) and were then incubated at 37°C/5% CO_2_ for a further 96 h. Cell viability assays were carried out using CellTiter-Glo^®^ Luminescent reagent as per the manufacturer’s instructions. Briefly, at the end of the 96 h incubation period, an equal volume of CellTiter-Glo^®^ reagent is added to each well and incubated at room temperature. The CellTiter-Glo^®^ reagent produces a luminescent signal which is proportional to the number of viable cells within the well. Luminescence was recorded using a Molecular Devices SpectramaxM3 plate reader and data were processed with GraphPad Prism using a four-parameter non-linear regression.

### *In vivo* Xenograft Studies

*In vivo* experiments were approved by the Animal Care and Use Committee of Oncodesign (OnComEt). Two Karpas-299 (T-anaplastic large cell lymphoma, ALCL) xenograft studies were performed in healthy female CB17-SCID mice (CBySmn.CB17-Prkdcscid/J, Charles River Laboratories) testing cyclodextrin ADCs (xenograft study 1) and crown ether ADCs (xenograft study 2) and relevant controls. The animals were maintained in SPF health status according to the FELASA guidelines in housing rooms under controlled environmental conditions. The mice were γ-irradiated (1.44 Gy, ^60^Co) 24–72 h prior to tumor cell injection. Tumors were induced by subcutaneous injection of 10^7^ Karpas-299 cells in 200 µL of RPMI 1640 into the right flank. Tumors were measured in two-dimensions (width (w) and length (L)) twice per week with calipers, and the volume was estimated using the formula:

$$Tumor\;Volume\left(mm^{3}\right)=\left(w^{2}\;\times \;L\right)/2$$

Treatment tolerability was assessed by bi-weekly body weight measurement and daily observation for clinical signs of treatment-related side effects. Mice were euthanized when a humane endpoint was reached (1,600 mm^3^ tumor volume) or after a maximum of 71 days post-tumor induction.

### Xenograft Study 1 (Cyclodextrin ADCs)

SCID mice with an average body weight of 17.5 g were used for cell inoculation (Day 0). Fourteen days after tumor implantation (Day 14), animals were randomized into groups of eight mice (200 mm^3^ mean tumor volume) and treatment was initiated. The animals from the vehicle group received a single intravenous (i.v.) injection of PBS. The treated groups were dosed with a single i.v. injection of ADCs **4d, 4b, 4f**, **6b** and Adcetris® at 0.5 mg/kg or 1 mg/kg.

### Xenograft Study 2 (Crown Ether ADCs)

SCID mice with an average body weight of 18.1 g were used for cell inoculation (Day 0). Twelve days after tumor implantation (Day 12), animals were randomized into groups of eight mice (211 mm^3^ mean tumor volume) and treatment was initiated. The animals from the vehicle group received a single i.v. injection of PBS. The treated groups were dosed with a single i.v. injection of ADCs 5a, 5b, 6a and 6b at 0.4 mg/kg or 0.8 mg/kg, or Adcetris® at 1 mg/kg.

## Results

### Linker-Payload and ADC Synthesis

**FIGURE 1:**
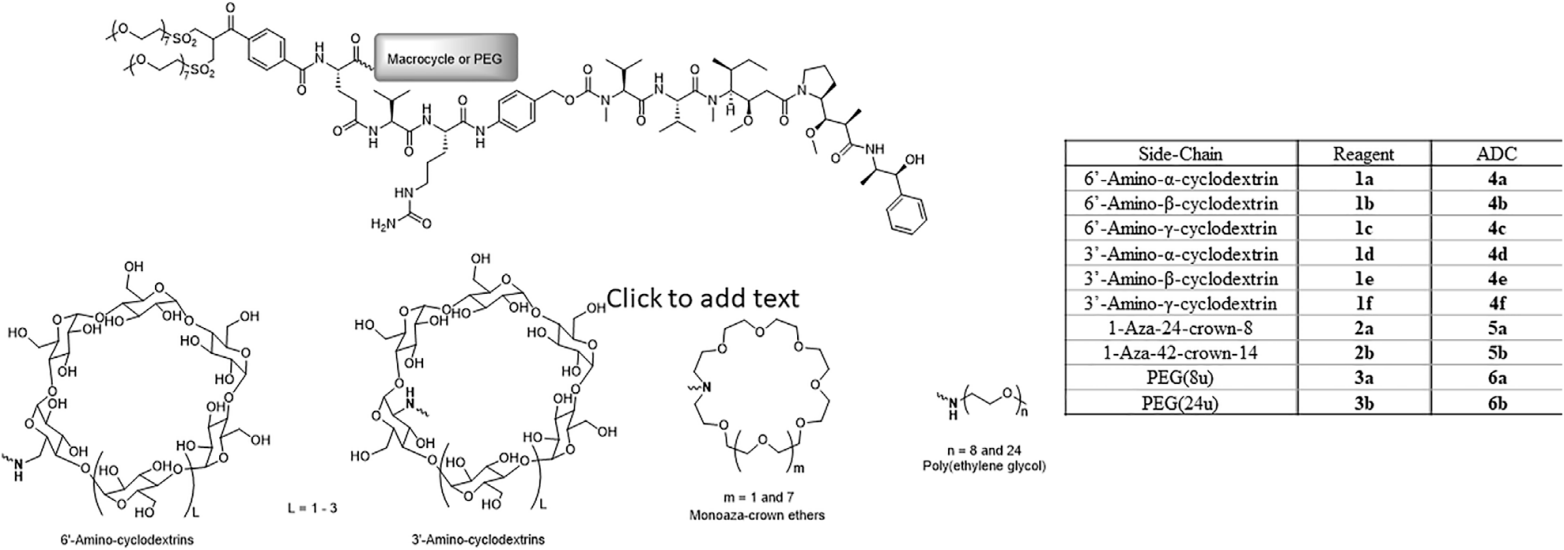
Structures of disulfide re-bridging reagents comprising glutamic acid-based branching points with linker-payload (Val-Cit-PAB-MMAE) and macrocycle (cyclodextrin or crown ether) or PEG appended.

Ten reagents with generic structures consisting of a *bis*-sulfone conjugating linker and a glutamic acid branching point, with the prodrug Val-Cit-PABC-MMAE and either a macrocycle or linear PEG chain appended were designed and synthesized (Figure 1). Six of the reagents (**1a-f**) contained α-, β- or γ-cyclodextrins and were linked to the reagent *via* 3′- or 6′-amino modifications on the macrocycles. Two 1-aza-crown ethers were prepared *via* macrocyclization of ethylene glycol derivatives to prepare 1-aza-24-crown-8 and 1-aza-42-crown-14. These 1-aza-crown ethers were successfully coupled to the branched template structure to yield reagents **2a-b**. As comparators, two structurally analogous reagents containing discrete PEGs containing 8 and 24 repeat units were also prepared. The 24-unit reagent (**3b**), included in this study as a reference, is a known reagent architecture and was previously conjugated to brentuximab variants to produce highly efficacious DAR 4 and DAR 6 ADCs (Pabst et al., 2017). Reagent **3a**, containing an 8-unit PEG side-chain, was synthesized to serve as a direct comparator to 24-crown-8 reagent **2a**.

Ten brentuximab ADCs with DAR 4 were prepared *via* an established disulfide re-bridging conjugation method (Bird et al., 2020) using cyclodextrin reagents **1a-f**, crown ether reagents **2a-b** and PEGylated reagents **3a-b**. Each crude conjugation reaction was purified by preparative HIC to isolate the DAR 4 ADC for biological evaluation.

### *In vitro* Cytotoxicity Assay

Prior to *in vivo* evaluation, the *in vitro* activity of each ADC was confirmed within a cell viability assay using CD30^+^ Karpas-299 cells. IC50 values obtained for each ADC are given in Table 1 (representative cell-viability assay curves are given in Supplementary Figure S1). All of the ADCs displayed sub-nanomolar potency (10^–11^ M range) similar to Adcetris^®^ (16 pM). In all cases, the ADCs displayed greater potency than the payload, MMAE.

**TABLE 1:** *In vitro* cell viability assay results for ADCs **4a-f**, **5a-b**, **6a-b**, Adcertis^®^ and MMAE in Karpas-299 cells. Average IC50 (mean) and standard deviations (SD) were determined from n ≥ 2 experiments.

ADC	IC50 (pM)	SD (pM)	ADC	IC50 (pM)	SD (pM)
**4a**	30	8	**5a**	30	14
**4b**	22	7	**5b**	34	15
**4c**	28	7	**6a**	32	13
**4d**	16	4	**6b**	19	5
**4e**	19	5	Adcetris®	16	13
**4f**	16	2	MMAE	124	118

The bold values refers to the structure of the ADCs.

### *In vivo* Xenograft Study 1

Following the similarities in potencies obtained from the *in vitro* cell viability assays, *in vivo* xenograft studies were performed to differentiate between the activities of these conjugates further. Within this first study, cyclodextrin-containing conjugates **4b**, **4d**, **4f** dosed at 0.5 or 1 mg/kg were compared with Adcetris^®^ dosed at 1 mg/kg, or conjugate **6b** dosed at 0.5 or 1 mg/kg. Animals were dosed on day 14 following tumor implantation when the tumor volume was approximately 200 mm^3^.

**FIGURE 2:**
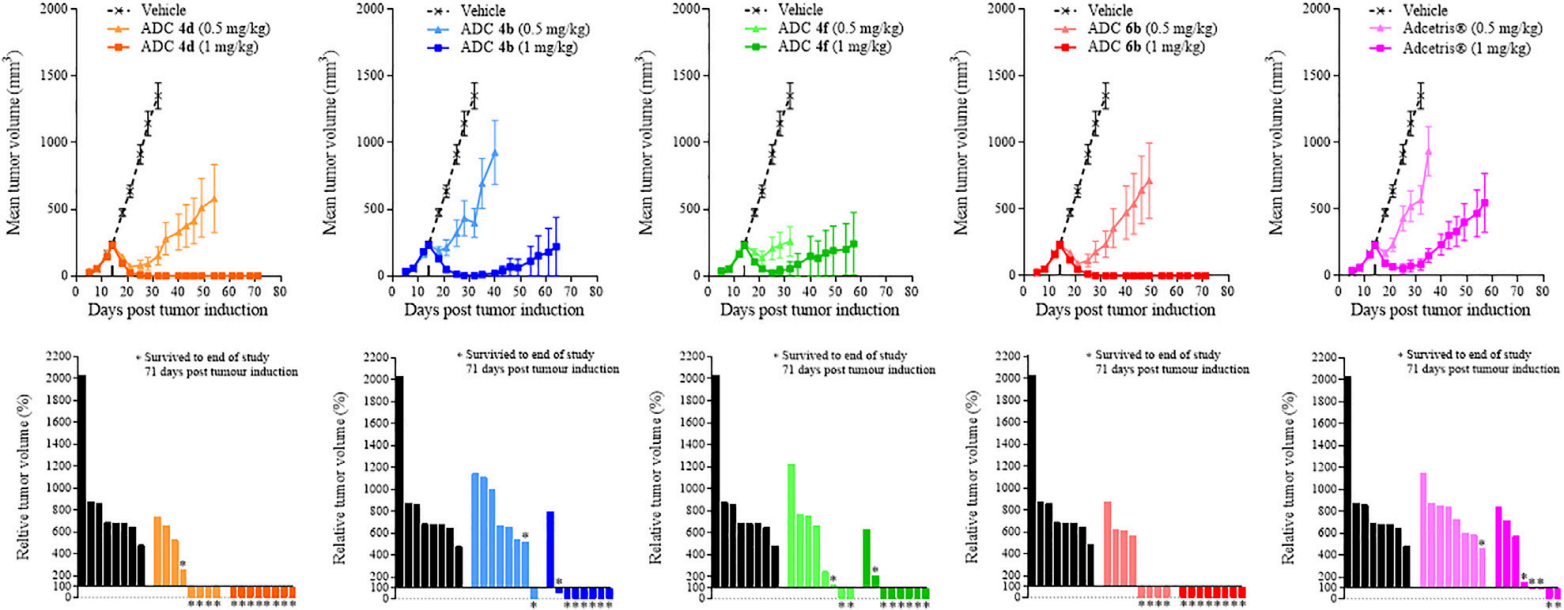
Tumor growth curves and relative tumor volume (%) plots taken on the final day of the study (Day 71) for xenograft study 1. Mean tumor volume was plotted until the first death in the group was recorded. Relative tumor volumes were calculated as percentage change on the final day of the study (Day 71) relative to the tumor volume on the day of treatment administration (Day 14).

Figure 2 shows *in vivo* xenograft mean (+/- SEM) and relative tumor volumes measured over time within mice dosed with either ADC **4b**, **4d**, **4f** and Adcetris® at 0.5 or 1 mg/kg. At 0.5 mg/kg dose, the conjugates reduce tumor growth relative to the vehicle control, but do not prevent tumor growth, as shown by the increasing tumor volumes throughout the duration of the study. ADCs **4d** and **4f** appear to slow tumor growth to a greater extent than ADC **4b** and the positive control comparator, Adcetris®, as shown more clearly by the waterfall plots. At 1 mg/kg dose, the effect of these ADCs upon tumor growth is much more striking, with conjugate **4d** showing a complete reduction of tumor volume down to 0 mm^3^ following dosing on day 14. This complete reduction of tumor growth was maintained throughout the duration of the study until Day 71. The inhibitory effects of conjugates **4b** and **4f** upon tumor growth were less pronounced than ADC **4d**, but nonetheless show a significant reduction in tumor growth throughout the duration of the study, displaying lower mean tumor volume values than the clinical comparator Adcetris® by the end of the study at day 71. The relative tumor volume (%) plots also show that 0 mm^3^ tumor volume (i.e. complete tumor regression) was achieved within 6 out of 8 animals for conjugates **4b** and **4f**, whereas only 2 out 8 animals measured 0 mm^3^ for the Adcetris® treated group. In comparison, for conjugate **4d**, complete tumor regression was achieved within 8 out of 8 animals, (0 mm^3^ final tumor volume), within the 1 mg/kg group and even 4 out of 8 animals achieved this for the 0.5 mg/kg group. Similar data were achieved with conjugate **6b**, which showed 1 less animal surviving to the end of the study compared with **4d**. These data show that conjugates **4d** and **6b** have comparable *in vivo* efficacies. All of these ADCs show greater *in vivo* efficacy over the clinical comparator Adcetris® within this tumor model. In addition to the tumor volume analyses, body weight measurements taken from each group show that all ADCs were well tolerated in comparison to the vehicle treated group (Supplementary Figure S2).

### *In vivo* Xenograft Study 2

**FIGURE 3:**
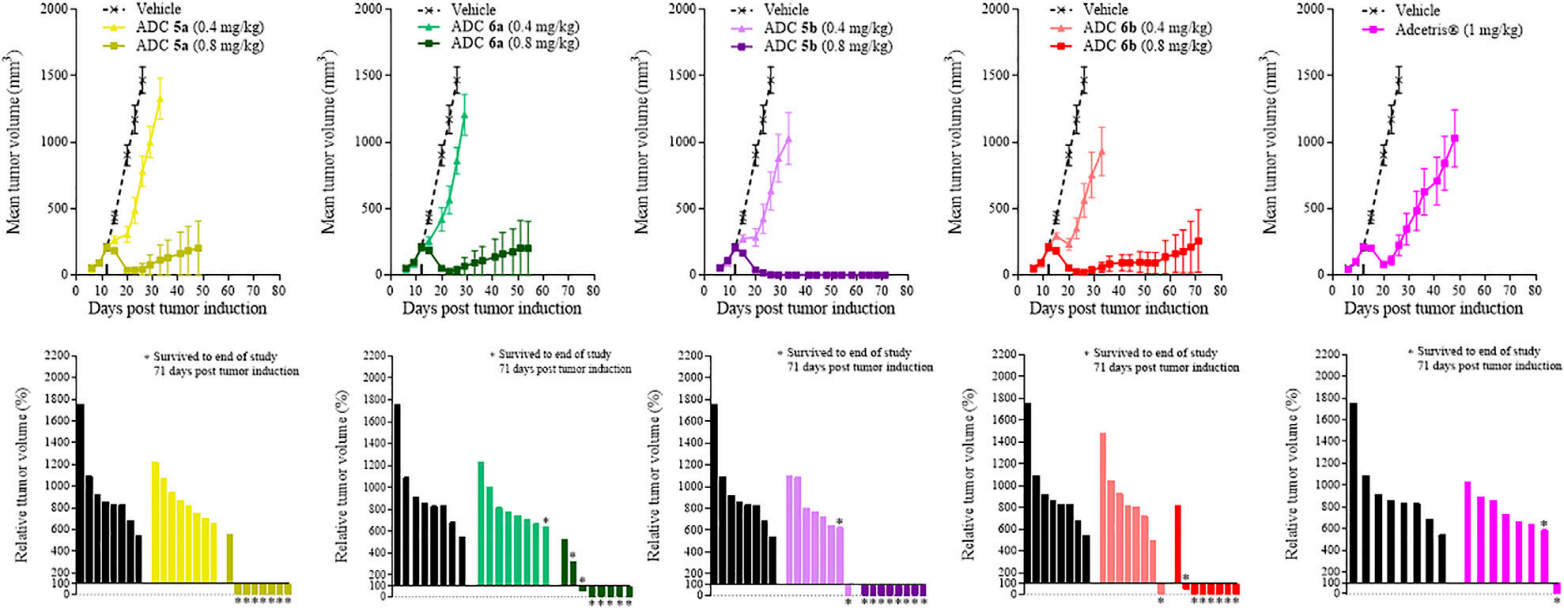
Tumor growth curves and relative tumor volume (%) plots taken on the final day of the study (Day 71) for xenograft study 2. Mean tumor volumes was plotted until the first death in the group was recorded. Relative tumor volumes were calculated as percentage change on the final day of the study (Day 71) relative to the tumor volumes on the day of treatment administration (Day 12).

Similarly to the cyclodextrin-containing conjugates, *in vivo* studies were also required to differentiate between the activities of the crown ether-containing conjugates, **5a** and **5b**, and their linear PEG-containing comparators **6a**, **6b** and Adcetris®. In contrast to Study 1, to differentiate the efficacies of these conjugates further, the doses of conjugates **5a**, **5b**, **6a** and **6b** were reduced to 0.4 and 0.8 mg/kg (Adcetris® dose was maintained at 1 mg/kg). Animals were dosed on day 12 following tumor implantation when the tumor volume was approximately 200 mm^3^. Figure 3 shows data obtained with the same *in vivo* xenograft tumor model as in Study 1, where tumor volumes were measured over time within mice dosed with ADCs **5a**, **5b**, **6a** or **6b** at 0.4 or 0.8 mg/kg. At 0.4 mg/kg, these ADCs had little effect upon tumor growth, following a similar growth rate to the vehicle control after Day 20 of the study. As with the previous study, animals were dosed with Adcetris® at 1 mg/kg and the tumor volumes measured over 71 days as a comparator. Adcetris® was given at Day 14 and showed an initial reduction in tumor volume, which was lost shortly after Day 20, resulting in a steady increase in tumor volume thereafter. Within this group, one animal out of the cohort of 8 achieved a complete reduction in tumor volume, where no tumor could be detected by the end of the study (also indicated by the relative tumor volume (%) plot within Figure 3). By comparison, the cohorts treated with ADCs **5a**, **5b**, **6a** and **6b** at 0.8 mg/kg dose each showed reductions in mean tumor volume for extended periods. Notably, the efficacy of conjugate **5b** was outstanding, achieving a mean tumor volume of 0 mm^3^, (i.e. complete tumor regression), for the duration of the study up to Day 71. The relative tumor volume (%) plots also show that conjugates **5a** and **5b** have the greatest efficacy, with 7 out of 8 and 8 out of 8 animals surviving tumor free respectively at 0.8 mg/kg dose, whereas for conjugates **6a** and **6b**, 5 out of 8 and 6 out of 8 animals survived tumor free at the end of the study respectively. As observed within Study 1 previously, the body weights of each animal were measured throughout and showed that the ADCs were well tolerated, as determined by comparison of the body weights of the treatment groups with the vehicle group, and no treatment related deaths were recorded across all groups (Supplementary Figure S3).

## Discussion

Cluster of differentiation 30 (CD30) is a type I transmembrane protein of the tumor necrosis factor receptor (TNFR) superfamily expressed on the medullary of the thymus gland and on a subset of activated T and B cells (Romagnani et al., 1998; van der Weyden et al., 2017). CD30 expression is not normally detected in healthy tissue outside of the immune system, however high levels of CD30 expression are found in Hodgkin lymphoma (HL) and ALCL, making it an attractive target for antibody-based immunotherapy. Numerous naked and conjugated CD30-targeting mAbs have entered clinical development but despite promising results from animal models, early candidates were unsuccessful (Younes, 2011; Schirrmann et al., 2014). Clinical success was eventually achieved with brentuximab vedotin (SGN-35), an ADC comprising a chimeric anti-CD30 IgG1 antibody conjugated to the potent cytotoxin, MMAE, *via* an enzymatically cleavable MC-Val-Cit-PABC linker. Conjugation of the linker-payload to the antibody is achieved over two steps: 1) partial reduction of the interchain disulfide bonds of the antibody followed by 2) thiol-maleimide conjugation to the unpaired cysteines liberated during reduction. The nature of the conjugation process results in a heterogeneous mixture of differently loaded species ranging from DAR 0 to DAR 8, with an average drug loading of approximately 4. Phase II clinical trials involving brentuximab vedotin as a single agent in patients with relapsed or refractory HL and ALCL resulted in objective response rates of 75 and 86% respectively (Pro et al., 2012; Younes et al., 2012). Based on these impressive results the FDA granted accelerated approval of brentuximab vedotin in 2011 and the ADC has since been marketed as Adcetris®.

Ongoing efforts to improve ADC efficacy have included focussing upon the design of linker-payload reagent architectures. In our previous work we investigated the inclusion of PEG in ADC reagent design, where structures containing branching architectures were revealed to enhance ADC efficacy over structures with no PEG component and where the PEG is included as a spacer within the ADC backbone (Pabst et al., 2017). Other studies have corroborated these results including research from Lyon and coworkers (Lyon et al., 2015; Burke et al., 2017). In the present work, we designed and synthesized eight new reagents using hydrophilic macrocycles in place of the linear PEG component. Each reagent contained identical *bis*-sulfone disulfide rebridging conjugating groups, glutamic acid branching points connecting Val-Cit-PABC-MMAE as linker-payload. The reagents designed varied only in the nature of the hydrophilic side-chain moiety appended to the branching point. Six new reagents containing cyclodextrins, two reagents for each of α-, β- and γ-cyclodextrin variants with mono-amino substituents in the 3′ or 6′ positions (Figure 1, reagents **1a-f**) were produced. Two further reagents bearing 1-aza-crown ethers were synthesized, the smaller macrocycle incorporated into the reagent template was a 24-crown-8 based ring and the larger macrocycle was a 42-crown-14 derivative (Figure 1, reagents **2a-b**). Two reagents with 8- and 24-unit linear PEG chains (Figure 1, reagents **3a-b**) were also synthesized so comparator ADCs could be prepared. Each of the ten disulfide rebridging reagents described were conjugated to brentuximab *via* reaction at pre-reduced interchain disulfide bonds to produce ten DAR 4 ADCs for biological evaluation. ADCs **4a-f** were prepared using the cyclodextrin based reagents, ADCs **5a-b** were synthesized using the crown ether reagents and the linear PEG reagents were used to prepare ADCs **6a-b**.

Table 1; Supplementary Figure S1 shows *in vitro* cell-viability data for the conjugates produced against Karpas-299 cells. Within each figure both Adcetris^®^ and conjugate **6b** were used as internal controls as comparators for the test ADCs. From Supplementary Figures S1A–S1C, it can be determined that the cell killing potency of each test ADC is higher than the free payload, MMAE, with each conjugate displaying similar IC50 values in the same range as conjugate **6b** and Adcetris^®^. These data suggest that although the *in vitro* assay is a reliable test for determining the overall activity of an ADC, as has been demonstrated in previous studies it may not be the best predictor of *in vivo* efficacy (Hamblett et al., 2004; Pabst et al., 2017).

Figure 2 shows that all of the cyclodextrin-containing ADCs display improved *in vivo* efficacy over the clinical comparator Adcetris^®^ within this Karpas-299 xenograft tumor model. These data were in contrast to the *in vitro* data obtained, which showed that all of the cyclodextrin conjugates were of similar or slightly poorer potency to Adcetris^®^. Figure 2 also shows that 3′-amino-α-cyclodextrin containing conjugate **4d** in particular had outstanding efficacy in this model, equivalent to that of the PEG containing conjugate **6b**. These data are very encouraging and supportive of future development of cyclodextrin-containing ADCs.

From Supplementary Figure S1C, it can be seen from the *in vitro* data that all of the PEG-containing ADCs; **5a**, **5b**, **6a** and **6b** have comparable IC50s to that of Adcetris^®^. Once again, this is not reflected within the *in vivo* efficacy results in Figure 3. It is notable that conjugates **5b** and **5a** are particularly efficacious *in vivo*, more so than Adcetris^®^ and even conjugate **6b**, which is again in contrast to the *in vitro* IC50 data. Conjugate **5b** appears to be the best of these ADCs, which as with the cyclodextrin-containing ADCs, is very encouraging for further development of crown-ether containing ADCs in the future.

## Conclusion

In conclusion, we tested whether hydrophilic macrocycles (cyclodextrin or crown ethers) may be used as hydrophilic substitutes for the polymeric element present in known ADC structures. Within current ADC structures, it is hypothesized that the linear polymer structure, imparts greater efficacy to ADCs *in vivo* by forming a ‘shield’ around the hydrophobic linker-payload structure, whereby the hydrophobicity of the conjugate is ‘masked’, resulting in a slower clearance of the ADC from the subject (Lyon et al., 2015). It may be considered therefore that in restricting the size and mobility of the polymeric element of the ADC, one may expect decreased shielding of the linker-payload and therefore poorer retention within the subject, resulting in reduced efficacy. However, we have shown that this is not the case for the cyclodextrin- or crown ether- containing ADCs tested in this work.

## Data Availability

The original contributions presented in the study are included in the article/Supplementary Material, further inquiries can be directed to the corresponding author.
