# Supplementary figures and images for: Incorporation of Hydrophilic Macrocycles Into Drug-Linker Reagents Produces Antibody-Drug Conjugates With Enhanced in vivo Performance
Source: Front Pharmacol. 2022 Jun 17;13:764540. doi: 10.3389/fphar.2022.764540 (PMC9247464; doi:10.3389/fphar.2022.764540)

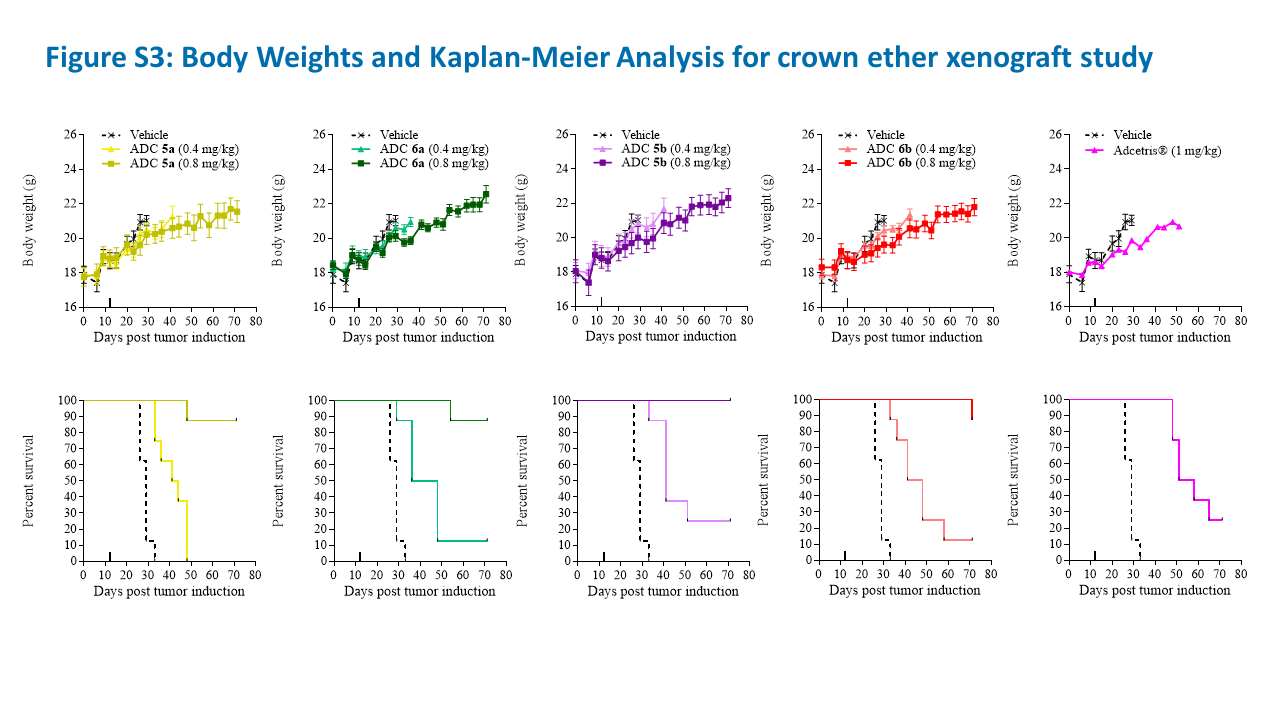

Supplement: Supplementary file 1 [file Image3.tif]

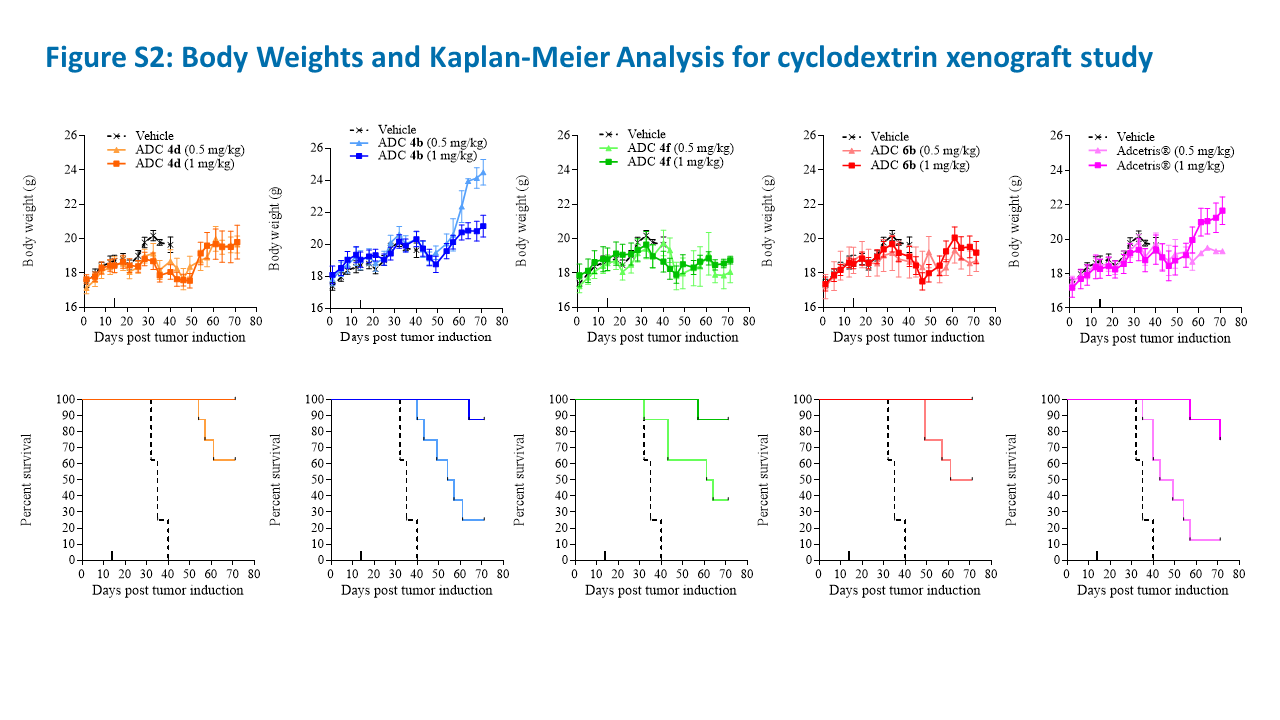

Supplement: Supplementary file 2 [file Image2.tif]

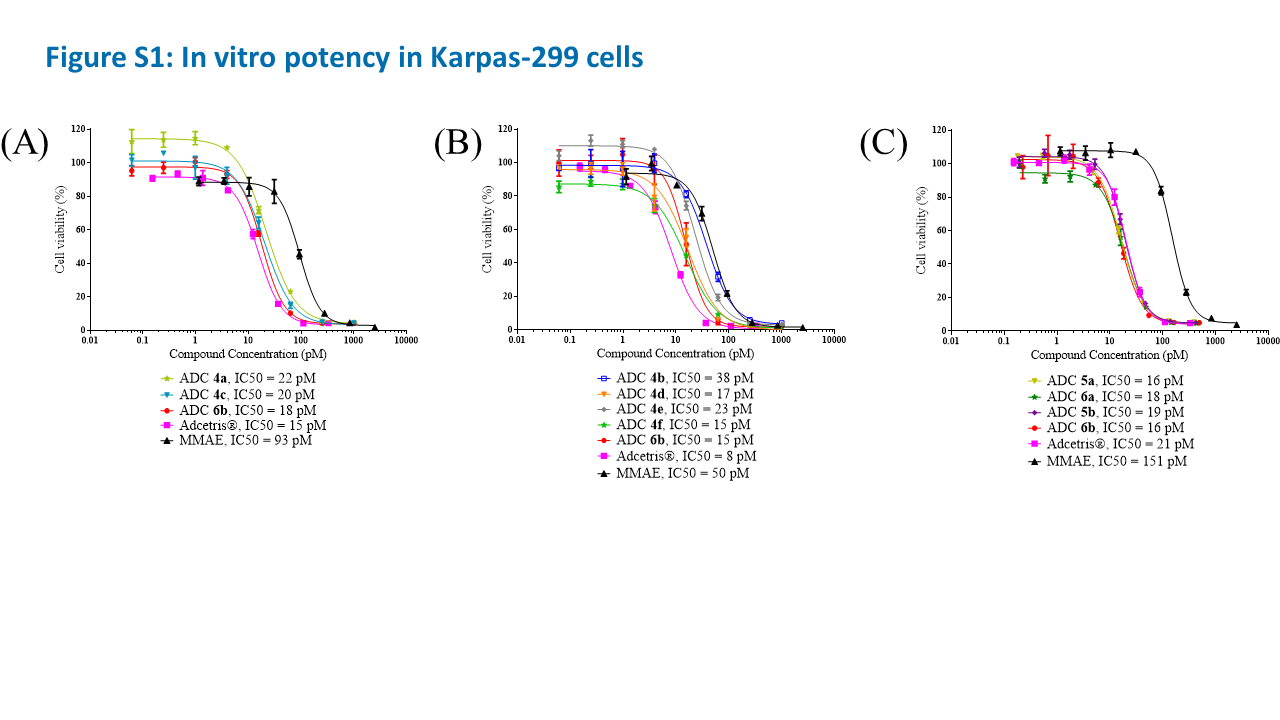

Supplement: Supplementary file 3 [file Image1.tif]
